# Challenges and Lessons from a Remote Intervention for Family Caregivers: A Feasibility Study of the EMPOWER Program

**DOI:** 10.1093/geroni/igaf122.3464

**Published:** 2025-12-31

**Authors:** Katie Trainum, Rosa Schnyer, Bo Xie

**Affiliations:** University of Pennsylvania, Philadelphia, Pennsylvania, United States; The University of Texas at Austin, Austin, Texas, United States; The University of Texas at Austin, Austin, Texas, United States

## Abstract

Remotely delivered interventions offer advantages like broader geographic reach and increased accessibility. However, they also introduce challenges, including scams in the recruitment and implementation as well as technological barriers. This paper reports challenges encountered and lessons learned through a feasibility study of EMPOWER (Engage your Mind and Body to Promote your Own Wellness, Energy and Relaxation), a self-care health intervention incorporating complementary and integrative techniques. In Fall 2024, we conducted four EMPOWER sessions (three via Zoom, one in-person). In the first two Zoom sessions, to ensure participants’ privacy and comfort, we did not require verbal or camera engagement, nor did we have a moderator present in the breakout room discussions. After the first two Zoom sessions, we discovered suspicious activity, including similar email formats, inconsistent demographics (i.e., higher-than-expected male and younger participants), and IP address anomalies (survey responses originating from outside the research area and multiple responses from single IP addresses). As a result, data collected from those first two Zoom sessions had to be discarded. Based on these experiences, we recommend several strategies for future virtual caregiving interventions. These include recruiting participants through established in-person groups; implementing rigorous participant vetting processes to verify authenticity; limiting responses to one per IP address; requiring verbal or camera engagement during the intervention sessions; and moderating Zoom breakout room discussions. While remotely delivered interventions present challenges, creative solutions can help maintain research integrity and enhance participant experience. Further work is needed to evaluate the efficacy of these strategies.

